# High waist circumference is a risk factor for hypertension in normal‐weight or overweight individuals with normal metabolic profiles

**DOI:** 10.1111/jch.14528

**Published:** 2022-06-23

**Authors:** Chen Cheng, Jin‐Yu Sun, Ying Zhou, Qi‐Yang Xie, Li‐Yuan Wang, Xiang‐Qing Kong, Wei Sun

**Affiliations:** ^1^ Cardiovascular Research Center The Affiliated Suzhou Hospital of Nanjing Medical University Suzhou Municipal Hospital Gusu School Nanjing Medical University Suzhou Jiangsu China; ^2^ Department of Cardiology The First Affiliated Hospital of Nanjing Medical University Nanjing Medical University Nanjing Jiangsu China; ^3^ Key Laboratory of Targeted Intervention of Cardiovascular Disease Collaborative Innovation Center for Cardiovascular Disease Translational Medicine Nanjing Medical University Nanjing Jiangsu China

**Keywords:** all‐cause mortality, body mass index, cardiometabolic profiles, hypertension, waist circumference

## Abstract

This study aims to investigate the relationship between waist circumference and hypertension risk in normal‐weight/overweight individuals with normal cardiometabolic profiles. The authors included 7217 normal‐weight and overweight individuals with normal cardiometabolic profiles from the 2001 to 2014 US National Health and Nutrition Examination Survey. The authors summarized demographic characteristics, cardiometabolic profiles, and behavioral factors across waist circumference quartiles. Then, in the logistic regression analysis, the authors observed a positive and significant association between waist circumference (as a continuous variable) and the prevalence of hypertension in all three models (nonadjusted, minimally adjusted, and fully adjusted), with odds ratios (95% confidence intervals) of 1.76 (1.65–1.86), 1.29 (1.20–1.39), and 1.24 (1.09–1.40), respectively. When analyzed as a categorical variable, individuals in the highest waist circumference group had a 1.48‐fold increased risk of hypertension than the lowest group in the fully adjusted model. Moreover, the Cox regression analysis revealed a positive and significant association between waist circumference and all‐cause mortality in individuals with hypertension in the nonadjusted model (HR, 1.27; 95% CI, 1.10–1.47) and the fully adjusted model (HR, 1.59; 95% CI, 1.22–2.06). In conclusions, our results showed that, even in those with normal metabolic profiles, high waist circumference was significantly associated with the increased prevalence of hypertension. And once hypertension has been established, patients with high waist circumference showed elevated all‐cause mortality. Therefore, waist circumference should be routinely measured and controlled regardless of metabolic profiles.

## 1 INTRODUCTION

Over the past five decades, obesity has become a growing global health problem. Obesity is closely associated with multiple chronic diseases and contributes to decreased life quality and expectancy.^1^, ^2^ Since body mass index (BMI) was first proposed in the 19th century, it has been the most widely and frequently used anthropometric parameter to define obesity. However, BMI alone is insufficient to accurately evaluate the obesity‐related risk^3^, ^4^ because individuals with similar BMI might show different body fat distribution and muscle mass.^5^, ^6^

Compared with BMI, waist circumstance is more closely associated with the absolute amount of abdominal fat.^7^ Accumulating studies have revealed that waist circumference was closely associated with multiple cardiovascular diseases and all‐cause mortality, with or without adjusting for BMI.^8^, ^9^ A recent consensus statement by the IAS and ICCR Working Group recommended routinely measuring waist circumference alongside BMI to classify obesity and identify the high‐risk obesity phenotype.^10^ In the previous studies, we reported a positive association of waist circumference with hypertension prevalence and cardiometabolic dysregulation regardless of BMI.^11^, ^12^

Obesity is usually characterized by multiple metabolic abnormalities, including lipid metabolism abnormalities (increased serum triglyceride [TG] and decreased HDL‐cholesterol [HDL‐C] concentrations) and glucose metabolism abnormalities (raised fasting plasma glucose [FPG] and insulin resistance).^13^, ^14^ These concomitant metabolic abnormalities are vital mediators of obesity‐related hypertension.^15^, ^16^ Interestingly, accumulating studies suggested that abdominal fat may contribute to the development of hypertension via nonmetabolic pathways (such as the activation of the sympathetic nervous system [SNS]^17^, ^18^ or the renin‐angiotensin‐aldosterone system [RAAS]^19^). However, few studies investigated the cardiovascular risk in metabolically healthy individuals. It remains unclear whether high waist circumference is a risk factor for hypertension in individuals with normal metabolic profiles.

Therefore, our study was designed to shed new light on the association between waist circumference and hypertension independently of metabolic factors.

## 2 METHODS

### 2.1 Data source and study population

The US National Health and Nutrition Examination Survey (NHANES) is a publicly available survey that collects the health and nutrition information of the representative US population every other year. The National Death Index (NDI) is a centralized database that provides vital mortality information, which helps to investigate the relationship between multiple health factors and mortality.

This study used the cross‐sectional data from seven consecutive cycles (2001–2002, 2003–2004, 2005–2006, 2007–2008, 2009–2010, 2011–2012, 2013–2014) of NHANES. The survival‐related follow‐up information was acquired from the NDI database, which records survival status from the date of medical examination to either death or censoring (until December 31, 2015).

**FIGURE 1:**
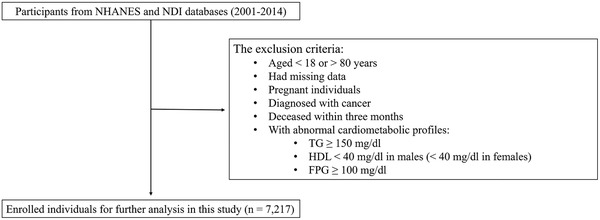
Flow chart of selection of eligible participants. NHANES: National Health and Nutrition Examination Survey. NDI: National Death Index

We included normal‐weight and overweight (defined as a BMI of 18.5–24.9 kg/m^2^ and 25.0–29.9 kg/m^2^, respectively) participants with multiple information, including body measurements, blood pressure, diabetes, smoking status, alcohol intake, dietary information, medical conditions, administration of antihypertensive, standardized biochemistry profiles and mortality information. The exclusion criteria were as follows (1) participants aged < 18 or > 80 years, (2) had missing data (BMI, waist circumference, or blood pressure records), (4) pregnant individuals, (5) diagnosed with cancer, (6) deceased within 3 months, (7) participants with abnormal cardiometabolic profiles (TG ≥ 150 mg/dl; HDL < 40 mg/dl in males, < 50 mg/dl in females; FPG ≥ 100 mg/dl) according to the definition of metabolic syndrome.^20^ Finally, a total of 7217 participants were enrolled (Figure 1). The analysis was approved by National Center for Health Statistics Research Ethics Review Board, and informed consent was acquired from all individuals.

### 2.2 Waist circumference measurement

According to the NHANES Anthropometry Procedures Manual, a trained health technician first instructed the participant to keep the appropriate posture and expose the measurement area (mainly the waist and hip area). Then, the technician would palpate the participant's hip area at the participant's right side to locate and mark the acme of the right iliac crest as the measurement level. Finally, the technician should measure the waist circumference with a tape measure horizontally surrounding the waist at the measurement level and lying snug but not compressing the skin at the end of the participant's normal expiration.

### 2.3 Definition of hypertension

Blood pressure was measured by the certified examiners trained by Shared Care Research and Education Consulting with sphygmomanometers, following the latest recommendations of the American Heart Association Human Blood Pressure Determination. After resting quietly in a seated position for 5 min and once the participant's maximum inflation level had been determined, three consecutive blood pressure readings were obtained, and we calculated the mean blood pressure.

In this study, individuals were defined as hypertensive when they met at least one of the following criteria: (1) mean systolic blood pressure ≥ 130 mm Hg, (2) mean diastolic blood pressure ≥ 80 mm Hg, (3) self‐reported hypertension, and (4) self‐reported usage of antihypertensive drugs.^21^, ^22^, ^23^

### 2.4 Demographic variables

Demographic variables were acquired from questionnaires, including age, sex, race (non‐Hispanic white, non‐Hispanic black, Mexican American, other Hispanic, and other races), income, and education (below high school, high school, and above high school). Poverty‐income ratio (PIR) was used to assess the income levels, which were classified as < 1.33, 1.33–3.50, and ≥ 3.50 recommended by the Supplemental Nutrition Assistance Program.^24^

### 2.5 Cardiometabolic and behavioral factors

Cardiometabolic and behavioral factors included body measurement, lipid profiles, blood glucose, sodium intake, cigarette/alcohol consumption, and history of cardiovascular diseases. Total‐to‐HDL cholesterol was calculated as total cholesterol divided by high‐density lipoprotein cholesterol.^25^ Diabetes was defined as self‐reported diabetes or hemoglobin A1c (HbA1c) ≥ 6.5%. More than 100 cigarettes consumption in life was determined as smoking, and participants who consumed ≥ 12 alcoholic drinks per year were considered alcohol users. Participants with self‐reported heart attack, angina pectoris, coronary heart disease, congestive heart failure, and/or stroke were considered to have a history of cardiovascular diseases.^26^ Details about the questionnaires and examinations protocols could be easily accessed on the NHANES website.

### 2.6 Statistical analysis

All the statistical analyses were performed and reported based on the recommendation from American Heart Association Scientific Publication Committee.^27^ We used multivariate multiple imputations to fill the missing covariates to maximize statistical power.^28^, ^29^ Kolmogorov–Smirnov test was used to assess the normality of the data distribution. Normally distributed continuous variables, non‐normally distributed continuous variables, and categorical variables were presented as mean ± standard deviation, median (Q1, Q3), and frequencies with percentages, respectively. The difference among waist circumference quartiles was evaluated by one‐way analysis of variance, the Kruskal–Wallis test, and the chi‐square test, respectively.

We applied a generalized linear model to evaluate the relationship between waist circumference and systolic blood pressure and analyzed their correlation by the Spearman correlation coefficient. Furtherly, we used the logistic regression to assess the association between waist circumference and hypertension, and odds ratios (ORs) with 95% confidence intervals (CIs) were calculated accordingly. In the minimally adjusted model, we adjusted for age, sex, race/ethnicity, education, HbA1c, smoking, drinking, PIR level, total‐to‐HDL cholesterol, and triglyceride level. In the fully adjusted model, we adjusted for BMI, age, sex, race/ethnicity, education, HbA1c, smoking, drinking, PIR level, total‐to‐HDL cholesterol, and triglyceride level. Moreover, we illustrated the relationship between waist circumference and hypertension by a restricted cubic spline with five knots (located at the 5^th^, 27.5^th^, 50^th^, 72.5^th,^ and 95^th^ percentiles), with the median waist circumference set as the reference.^30^

Furthermore, we adopted the Cox regression analysis to assess the association between waist circumference and all‐cause mortality in individuals with hypertension. The hazard ratios (HRs) with 95% CIs of the three models were calculated. In the minimally adjusted model, we adjusted for age, sex, race/ethnicity, education, HbA1c, smoking, drinking, PIR level, total‐to‐HDL cholesterol, and triglyceride level. BMI was additionally adjusted for in the fully adjusted model. We defined statistical significance as *P* of < .05. All statistical analyses were performed by the R software (version 3.6.1; R Foundation for Statistical Computing, Vienna).

## 3 RESULTS

### 3.1 Characteristics of the study population

Demographic characteristics, cardiometabolic profiles, and behavioral factors are summarized in Table 1. Compared with the individuals with low waist circumference, those with high waist circumference were older, less educated, and more were males, while the PIR level showed no significant difference. In the overall population, the prevalence of hypertension was 24.1%, the median age was 41.0 (29.0–55.0) years, the median BMI was 24.5 (22.2–26.8) kg/m^2^, and the median waist circumference was 87.1 (80.0–94.0) cm. Interestingly, although all the individuals had normal metabolic profiles (TG, HDL‐C, and FPG), those with higher waist circumference had higher total‐to‐HDL cholesterol, TG, and FPG levels. Moreover, they also showed a higher prevalence of hypertension and self‐reported cardiovascular diseases.

**TABLE 1:** Demographic characteristics, cardiometabolic profiles, and behavioral factors by waist circumference quartiles

	Overall	Q1 [61.3, 80.0]	Q2 (80.0, 87.1]	Q3 (87.1, 94.0]	Q4 (94.0, 120]	*P*‐value
N	7217	1814	1816	1824	1763	
Age (years), (median [Q1, Q3])	41.0 [29.0, 55.0]	32.0 [24.0, 45.0]	38.0 [28.0, 51.0]	43.0 [32.0, 57.0]	51.0 [39.0, 65.0]	< .001
Sex (Male), *n* (%)	3542 (49.1)	604 (33.3)	770 (42.4)	955 (52.4)	1213 (68.8)	< .001
Race/ethnicity, *n* (%)						< .001
Non‐Hispanic White	3432 (47.6)	797 (43.9)	835 (46.0)	835 (45.8)	965 (54.7)	
Non‐Hispanic Black	1561 (21.6)	413 (22.8)	382 (21.0)	420 (23.0)	346 (19.6)	
Mexican American	1033 (14.3)	205 (11.3)	251 (13.8)	301 (16.5)	276 (15.7)	
Other Hispanic	533 (7.4)	126 (6.9)	153 (8.4)	139 (7.6)	115 (6.5)	
Other races	658 (9.1)	273 (15.0)	195 (10.7)	129 (7.1)	61 (3.5)	
Education levels, *n* (%)						< .001
Below high school	1517 (21.0)	325 (17.9)	356 (19.6)	413 (22.6)	423 (24.0)	
High School	1544 (21.4)	364 (20.1)	391 (21.5)	387 (21.2)	402 (22.8)	
Above high school	4156 (57.6)	1125 (62.0)	1069 (58.9)	1024 (56.1)	938 (53.2)	
PIR level, *n* (%)						.053
< 1.33	1857 (25.7)	484 (26.7)	497 (27.4)	457 (25.1)	419 (23.8)	
1.33 to < 3.5	2349 (32.5)	599 (33.0)	595 (32.8)	599 (32.8)	556 (31.5)	
≥3.5	3011 (41.7)	731 (40.3)	724 (39.9)	768 (42.1)	788 (44.7)	
BMI (kg/m^2^), (median [Q1, Q3])	24.5 [22.2, 26.8]	21.1 [20.0, 22.4]	23.5 [22.3, 24.9]	25.6 [24.2, 27.1]	27.7 [26.3, 28.9]	<.001
Total‐to‐HDL cholesterol (median [Q1, Q3])	3.0 [2.6, 3.6]	2.7 [2.4, 3.2]	3.0 [2.5, 3.5]	3.2 [2.7, 3.8]	3.4 [2.8, 4.0]	<.001
Triglycerides (mg/dL), (median [Q1, Q3])	81.0 [60.0, 106.0]	69.0 [53.0, 92.0]	77.0 [58.0, 102.0]	85.0 [64.0, 110.0]	93.0 [71.5, 117.0]	<.001
FPG (mg/dL), (median [Q1, Q3])	87.0 [81.0, 92.0]	84.0 [79.0, 89.0]	86.0 [81.0, 91.0]	88.0 [82.0, 92.0]	89.0 [84.0, 94.0]	<.001
HbA1c (median [Q1, Q3])	5.3 [5.1, 5.5]	5.2 [5.0, 5.4]	5.3 [5.1, 5.5]	5.3 [5.1, 5.6]	5.4 [5.2, 5.6]	<.001
Hypertension (Yes, %)	1738 (24.1)	256 (14.1)	336 (18.5)	468 (25.7)	678 (38.5)	<.001
SBP (mm Hg), (median [Q1, Q3])	115.3 [107.3, 126.7]	111.3 [104.0, 120.7]	114.0 [106.0, 123.3]	116.7 [108.7, 128.0]	121.3 [112.7, 132.7]	<.001
DBP (mm Hg), (median [Q1, Q3])	69.3 [62.7, 76.0]	67.3 [61.3, 74.0]	68.7 [62.0, 75.3]	70.0 [64.0, 76.7]	72.0 [64.3, 78.7]	<.001
Cardiovascular diseases (Yes, %)	335 (4.6)	35 (1.9)	69 (3.8)	85 (4.7)	146 (8.3)	<.001
Smoking (Yes, %)	3039 (42.1)	681 (37.5)	727 (40.0)	758 (41.6)	873 (49.5)	<.001
Drinking (Yes, %)	798 (11.1)	200 (11.0)	212 (11.7)	198 (10.9)	188 (10.7)	.787
Diabetes (Yes, %)	152 (2.1)	12 (.7)	22 (1.2)	36 (2.0)	82 (4.7)	<.001

Abbreviations: PIR, poverty‐income ratio; BMI, body mass index; FPG, fasting plasma glucose; HbA1c, hemoglobin A1c; SBP, systolic blood pressure; DBP, diastolic blood pressure.

**FIGURE 2:**
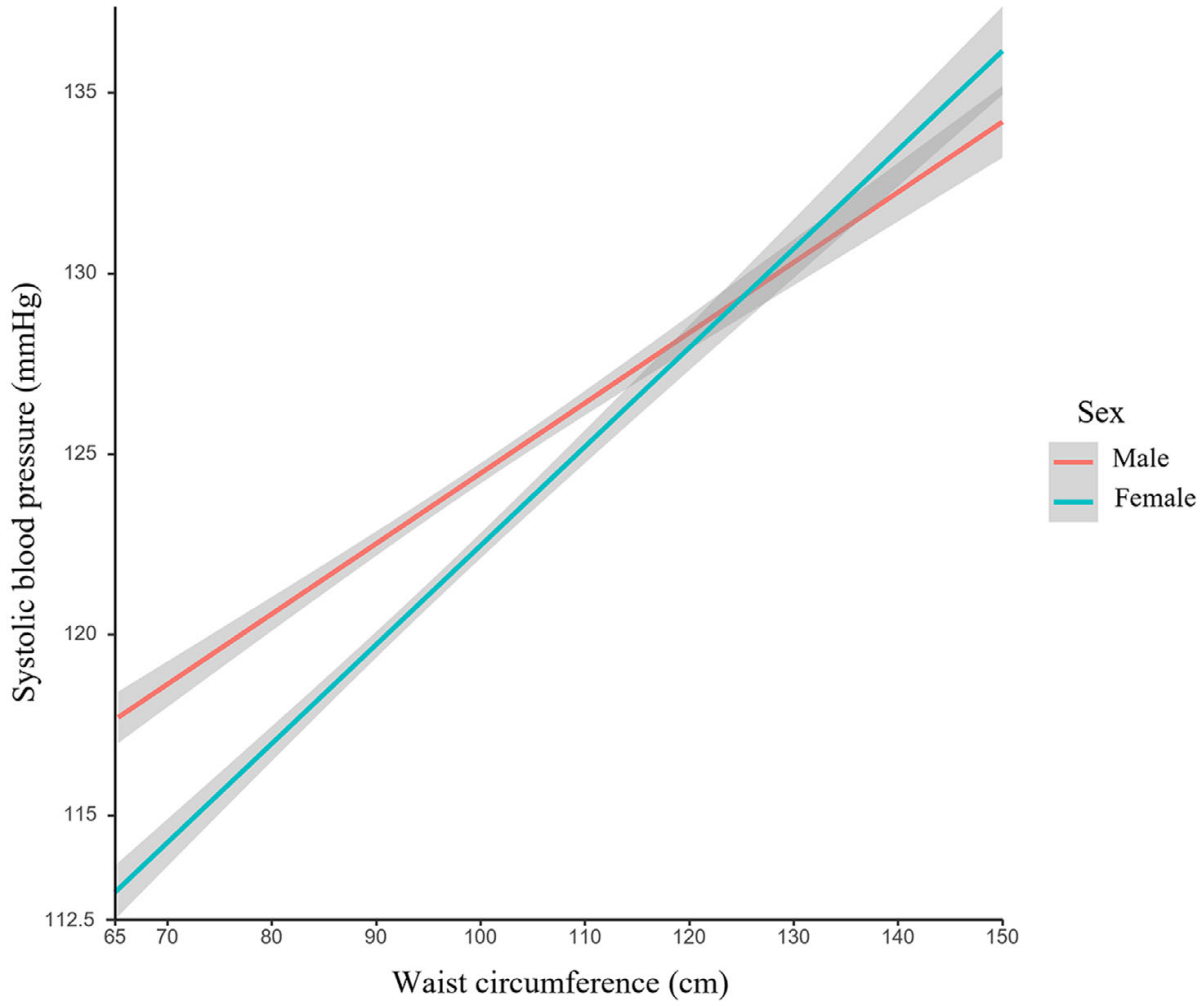
The fitted curve on the relationship between waist circumference and systolic blood pressure by the generalized linear model in males and females

As shown in Figure 2, waist circumference stratified by sex was positively and significantly associated with SBP, with a more considerable slope value observed in females.

### 3.2 The association between waist circumference and hypertension

Table 2 shows the association between waist circumference and hypertension. We observed a positive and significant association between waist circumference (as a continuous variable) and the prevalence of hypertension in all three models (nonadjusted, minimally adjusted, and fully adjusted), with ORs (95% CIs) of 1.76 (1.65–1.86), 1.29 (1.20–1.39), and 1.24 (1.09–1.40), respectively.

**TABLE 2:** The association of waist circumference with hypertension prevalence using logistic regression models

	Non‐adjusted model		Minimally‐adjusted model		Fully‐adjusted model	
	Odds ratio	*P*‐value	Odds ratio	*P*‐value	Odds ratio	*P*‐value
Waist circumference (Per 10 cm)	1.76 (1.65–1.86)	<.001	1.29 (1.20–1.39)	<.001	1.24 (1.09–1.40)	<.001
Categories						
Q1[61.3, 80.0]	Reference		Reference		Reference	
Q2(80.0, 87.1]	1.38 (1.16–1.65)	<.001	1.07 (.88–1.3)	.513	1.00 (.80–1.24)	.974
Q3(87.1, 94.0]	2.10 (1.78–2.49)	<.001	1.31 (1.08–1.59)	.007	1.15 (.90–1.47)	.262
Q4(94.0, 120]	3.80 (3.23–4.48)	<.001	1.79 (1.47–2.18)	<.001	1.48 (1.09–1.99)	.011

Minimally adjusted model: We adjusted for age, sex, race/ethnicity, education, HbA1c, smoking, drinking, PIR level, total‐to‐HDL cholesterol, and triglyceride level.

Fully adjusted model: We adjusted for BMI, age, sex, race/ethnicity, education, HbA1c, smoking, drinking, PIR level, total‐to‐HDL cholesterol, and triglyceride level.

Abbreviations: HbA1c, hemoglobin A1c; PIR, poverty‐income ratio; BMI, body mass index.

**FIGURE 3:**
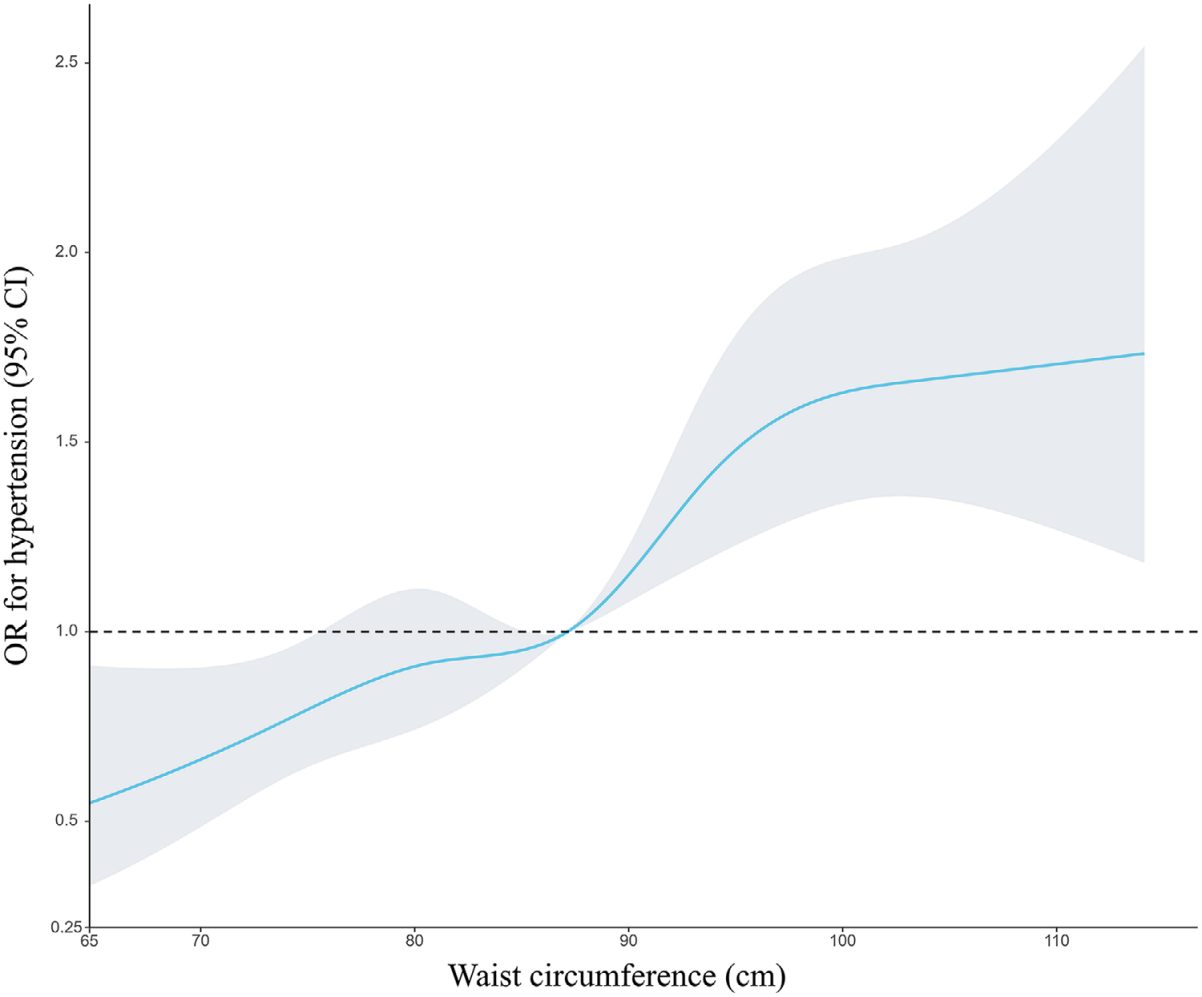
The adjusted restricted cubic spline model on the association between waist circumference and hypertension in normal‐weight and overweight individuals with normal cardiometabolic profiles. The median waist circumference of 87.1 cm was set as the reference. Multiple covariates were adjusted in the model, including age, race/ethnicity, sex, education level, diabetes, smoking status, alcohol consumption, PIR level. CI: Confidence interval; OR: Odds ratio; PIR: Poverty‐income ratio

When analyzed as a categorical variable, individuals in the highest waist circumference group had a 1.48‐fold increased risk of hypertension than those in the lowest group in the fully adjusted model. Furthermore, we used a restricted cubic spline to visualize the association between waist circumference and hypertension. An elevated risk of hypertension was observed with the increasing waist circumference levels after adjusting for multiple covariates (Figure 3).

### 3.3 The association between waist circumference and all‐cause death in hypertensive individuals

**FIGURE 4:**
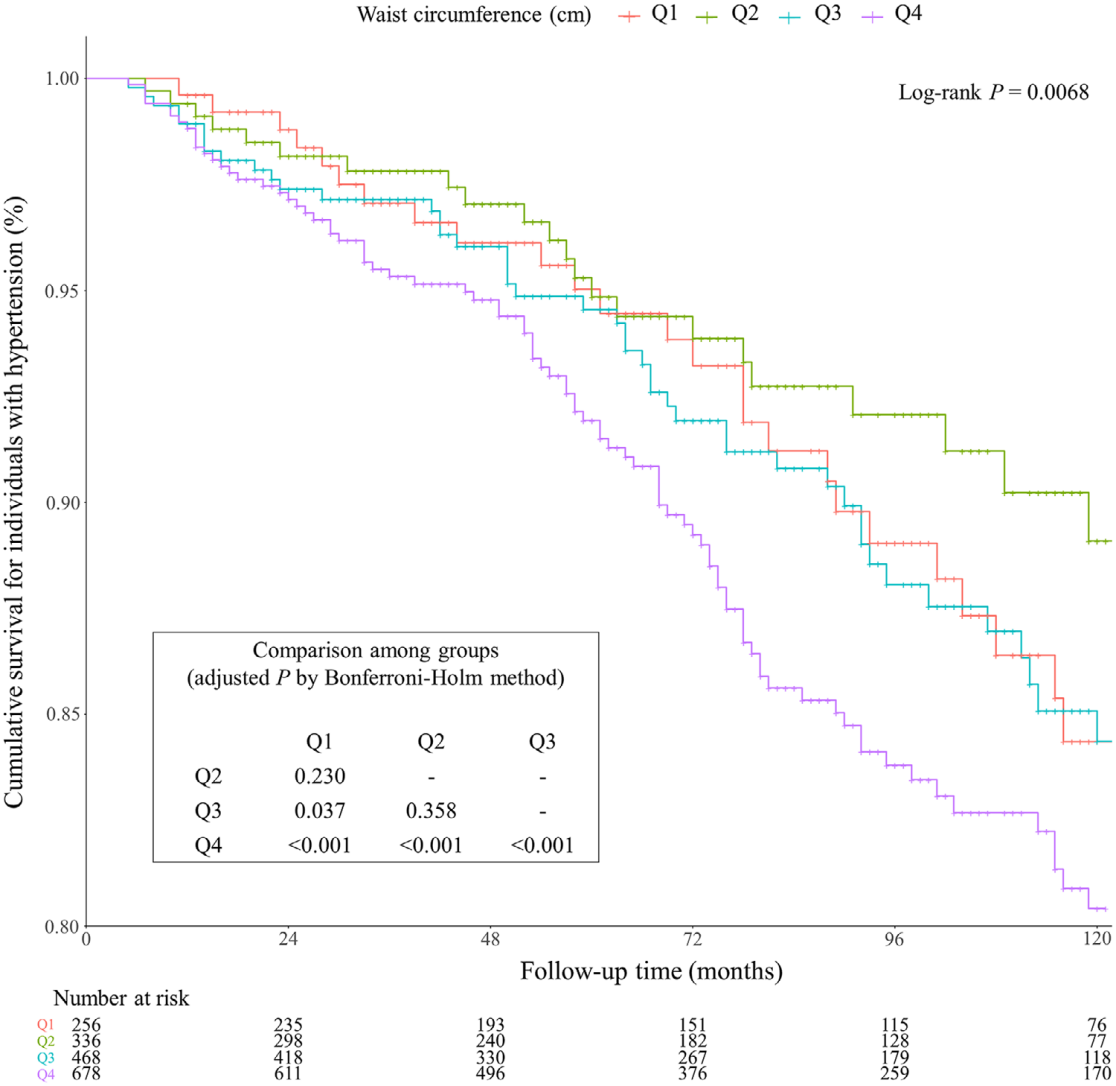
Kaplan–Meier plots in individuals with hypertension by waist circumference quintiles. The survival comparison among groups was adjusted by the Bonferroni–Holm method. Waist circumference quintiles were acquired from all individuals with hypertension. Q1: 61.3–80.0 cm; Q2: 80.0–87.1 cm; Q3: 87.1–94.0 cm; Q4: 94.0‐120 cm

As presented in Figure 4, Kaplan–Meier curve analysis revealed a significant difference in all‐cause mortality among waist circumference quartiles (Q1, Q2, Q3, and Q4) in individuals with hypertension (log‐rank *P* < .0001). Hypertensive individuals with high waist circumference showed significantly elevated all‐cause mortality than the low waist circumference groups. However, no significant difference was observed among hypertensive individuals with low levels of waist circumference (Q1 *vs*. Q2, *P* = .230; Q2 *vs*. Q3, *P* = .358).

The Cox regression analysis showed that waist circumference was significantly associated with all‐cause death in hypertensive individuals in the nonadjusted model (HR, 1.27; 95% CI, 1.10–1.47) and the fully adjusted model (HR, 1.59; 95% CI, 1.22‐2.06) (Table 3).

**TABLE 3:** The association of waist circumference with all‐cause mortality using Cox regression model

	Non‐adjusted model		Minimally‐adjusted model		Fully‐adjusted model	
	Hazard Ratio	*P*‐value	Hazard Ratio	*P*‐value	Hazard Ratio	*P*‐value
Waist circumference (Per 10 cm)	1.27 (1.10–1.47)	.001	1.02 (.87–1.20)	.775	1.59 (1.22–‐2.06)	.001

Minimally adjusted model: We adjusted for age, sex, race/ethnicity, education, HbA1c, smoking, drinking, PIR level, total‐to‐HDL cholesterol, and triglyceride level.

Fully adjusted model: We adjusted for BMI, age, sex, race/ethnicity, education, HbA1c, smoking, drinking, PIR level, total‐to‐HDL cholesterol, and triglyceride level.

Abbreviations: HbA1c, hemoglobin A1c; PIR, poverty‐income ratio; BMI, body mass index.

## 4 DISCUSSION

This study revealed the association between waist circumference and hypertension independently of metabolic factors based on normal‐weight and overweight individuals with normal cardiometabolic profiles from the 2001 to 2014 NHANES. In the logistic regression analysis, waist circumference (as a continuous variable) had a consistent and significant association with increased prevalence of hypertension (adjusted OR, 1.24; 95% CI, 1.09–1.40), and a similar result was observed in the restricted cubic spline. When analyzed as a categorical variable, the highest waist circumference group (94.0–120 cm), with a hypertension prevalence of 38.5%, had a 1.48‐fold higher risk than the lowest quartile group (61.3–80.0 cm). Moreover, the Cox regression analysis showed that hypertensive individuals with high waist circumference had significantly elevated all‐cause mortality (adjusted HR, 1.59; 95% CI, 1.22–2.06). These results suggested that waist circumference might be a risk factor for the occurrence and development of hypertension, independently of metabolic profiles.

Obesity, as a global health problem, has brought a considerable social burden for its close association with multiple chronic diseases and its contribution to a decreased quality and expectancy of life ^1,^.^31^ Although BMI has been widely used for defining obesity and has been observed to have a U/J‐shaped relationship with cardiovascular disease‐specific mortality and all‐cause mortality,^32^, ^33^ its inability to reflect body fat distribution has limited its application in evaluating obesity‐related cardiovascular risk because of the heterogeneous nature of obesity, in which visceral fat has a closer association with metabolic abnormalities compared with subcutaneous fat.^34^, ^35^ To our best knowledge, waist circumference is closely associated with the absolute quantity of abdominal fat and has unique advantages in assessing abdominal fat distribution.^7^, ^35^ Moreover, a recent consensus statement has highlighted the importance of routine measurement of waist circumference alongside BMI in classifying obesity and identifying individuals with high obesity‐related risk.^10^ Also, our previous studies have revealed that waist circumference is positively and significantly associated with hypertension prevalence and cardiometabolic dysregulation regardless of BMI.^11^, ^12^

Multiple studies have demonstrated that obesity is related to hypertension. Still, to the best of our knowledge, the specific mechanisms of the association of abdominal obesity with hypertension are complex and unclear, involving multiple dietary, genetic, epigenetic, and environmental factors.^36^, ^37^ Previous studies have revealed that concomitant cardiometabolic abnormalities are vital mediators of obesity‐related hypertension.^15^, ^16^, ^38^ Also, cardiometabolic risk factors was considered to play a mediation role between high waist circumference and increased morbidity and mortality risk.^10^, ^39^ Interestingly, accumulating studies suggested that abdominal fat may contribute to the development of hypertension via nonmetabolic pathways (such as the activation of SNS^17^, ^18^ or RAAS^19^). In this study, we observed a positive and significant association between waist circumference and prevalence of hypertension in the normal‐weight and overweight individuals with normal cardiometabolic profiles (adjusted OR, 1.24; 95% CI, 1.09–1.40). This study suggested that waist circumference might be a risk factor for hypertension independently of the above cardiometabolic profiles and supposed that, before the metabolic pathways and the BMI‐defined obesity occurred, the excessive abdominal fat represented by high waist circumference might have contributed to the occurrence of hypertension by nonmetabolic pathways. Moreover, once hypertension has been established, patients with high waist circumference show elevated all‐cause mortality (adjusted HR, 1.59; 95% CI, 1.22–2.06), which suggested that the nonmetabolic pathways might also play a role in the progression and prognosis of hypertension.

To data, multiple studies have revealed that SNS activation, especially the renal sympathetic nerves activity (RSNA), plays a vital role in obesity‐induced hypertension^17,^.^18^, ^38^, ^40^ And accumulating evidence has suggested that abdominal visceral fat had a positive and significant association with SNS activity, while such a relation was not evident in subcutaneous obese individuals.^41^, ^42^ Notably, some possible mediators of obesity‐related SNS activation have been suggested, such as adipokines (like leptin, angiotensinogen, interleukin‐6, and tumor necrosis factor‐α), angiotensin II, hyperinsulinemia, impaired baroreceptor reflexes, and activated chemoreceptor reflexes.^18^ For example, angiotensinogen can be secreted by adipocytes and is a crucial factor for the formation of angiotensin II. Notably, multiple studies have suggested that the secretion of angiotensinogen and the expression of related genes are greater in visceral compared with subcutaneous fat tissues.^19^, ^43^, ^44^, ^45^ And accumulating evidence has revealed that angiotensin II can increase SNS activity in animals^46^ and humans.^47^ Additionally, angiotensinogen released by adipose tissue can also activate the RAAS^19^ and stimulate the adrenal release of aldosterone, which regulates blood pressure by mineralocorticoid receptors in the vascular and renal systems.^48^ Interestingly, recent studies have suggested that metabolically healthy obesity phenotype may be a transient state,^49^, ^50^ which means individuals with high waist circumference but normal cardiometabolic profiles may transition to metabolically unhealthy with abnormal profiles in the future. Therefore, more molecular biological experimental studies are required to confirm and clarify the specific mechanisms underlying the association between abdominal fat and hypertension.

In this study, although all individuals are normal‐weight/overweight with normal cardiometabolic profiles, the high waist circumference groups, compared with the low waist circumference groups, still have a significantly higher prevalence of hypertension and higher all‐cause mortality once hypertension has been established. Therefore, we highlight the importance of routinely measuring waist circumference in evaluating obesity‐related cardiovascular risk and identifying high‐risk individuals, regardless of metabolic profiles. For metabolically healthy people without hypertension, routine measurement and early behavior or therapeutic intervention is beneficial for the primary prevention of hypertension. And for hypertensive patients, routine measurement and early intervention might decrease the all‐cause mortality and improve prognosis.

## 5 LIMITATIONS

Although our studies have several strengths in a nationally US sample (from NHANES and NDI databases), unique study design, and standard statistical analysis, some limitations should be mentioned. First, the individuals we included were limited to the US population, while data from other countries like China were lacking. Considering differences in fat distribution among races, the generalizability of our results should be further improved. Second, this study did not distinguish subtypes of hypertension (essential hypertension and secondary hypertension). And their mechanisms of occurrence and development differ significantly, in which waist circumference might play distinct roles. Third, we used TG, HDL, and FBG as representative cardiometabolic profiles based on the definition of metabolic syndrome, while cardiometabolic profiles are more than the three ones above. In our further studies, stricter criteria of normal cardiometabolic profiles are required for more accurate and reliable results. Fourth, although we adjusted for many known covariates in the statistical analysis, potential and unknown confounders may still exist. Last but not least, although our study revealed the association between waist circumference and hypertension prevalence, it is difficult to determine causality due to the inherent nature of a cross‐sectional study. More prospective studies are required to prove whether high waist circumference would cause a high risk of hypertension in individuals with normal metabolic profiles.

## 6 CONCLUSIONS

Our results showed that, even in those with normal metabolic profiles, high waist circumference was significantly associated with the increased prevalence of hypertension. And once hypertension has been established, patients with high waist circumference showed elevated all‐cause mortality. Therefore, waist circumference should be routinely measured and controlled regardless of metabolic profiles.

## AUTHOR CONTRIBUTIONS

Conception and design: Chen Cheng, Jin‐Yu Sun, Xiang‐Qing Kong, Wei Sun. Administrative support: Xiang‐Qing Kong, Wei Sun. Provision of study materials or patients: Chen Cheng, Jin‐Yu Sun, Qi‐Yang Xie. Collection and assembly of data: Chen Cheng, Jin‐Yu Sun, Li‐Yuan Wang. Data analysis and interpretation: Chen Cheng, Jin‐Yu Sun, Ying Zhou. Manuscript writing: All authors. Final approval of manuscript: All authors.

## CONFLICTS OF INTEREST

The authors declared no conflict of interest.
